# Supplementary material for: Service network design for freight railway transportation: The Chinese case
Source: PLoS One. 2022 Dec 1;17(12):e0276671. doi: 10.1371/journal.pone.0276671 (PMC9714731; doi:10.1371/journal.pone.0276671)
Supplement: Attachment — Submitted filename: Response to Reviewers.docx [file pone.0276671.s001.docx]

**Dear Prof. Dragan Pamucar for our paper PONE-D-22-11121:**

The authors sincerely thank you for the comments and suggestions which contribute to enhancing the quality of the paper. We have addressed the editor’s and reviewers’ comments and suggestions as well as the journal requirements in the revised paper. Each comment or suggestion is shown below in *Italic* followed with our action to modify the paper accordingly.

***Addressing the Journal Requirements***

***1. Please ensure that your manuscript meets PLOS ONE's style requirements, including those for file naming. The PLOS ONE style templates can be found at***

[***https://journals.plos.org/plosone/s/file?id=wjVg/PLOSOne_formatting_sample_main_body.pdf***](https://journals.plos.org/plosone/s/file?id=wjVg/PLOSOne_formatting_sample_main_body.pdf) ***and***

[***https://journals.plos.org/plosone/s/file?id=ba62/PLOSOne_formatting_sample_title_authors_affiliations.pdf***](https://journals.plos.org/plosone/s/file?id=ba62/PLOSOne_formatting_sample_title_authors_affiliations.pdf)

Thank you for your advice. We have modified the format as per the style requirements of PLOS ONE, including those for file naming.

***2. Thank you for stating the following in your Competing Interests section:***

***“NO authors have competing interests”***

***Please complete your Competing Interests on the online submission form to state any Competing Interests. If you have no competing interests, please state "The authors have declared that no competing interests exist.", as detailed online in our guide for authors at*** [***http://journals.plos.org/plosone/s/submit-now***](http://journals.plos.org/plosone/s/submit-now)

***This information should be included in your cover letter; we will change the online submission form on your behalf.***

Thank you for your advice. We have stated “The authors have declared that no competing interests exist.” in our revised cover letter.

***3. PLOS requires an ORCID iD for the corresponding author in Editorial Manager on papers submitted after December 6th, 2016. Please ensure that you have an ORCID iD and that it is validated in Editorial Manager. To do this, go to ‘Update my Information’ (in the upper left-hand corner of the main menu), and click on the Fetch/Validate link next to the ORCID field. This will take you to the ORCID site and allow you to create a new iD or authenticate a pre-existing iD in Editorial Manager. Please see the following video for instructions on linking an ORCID iD to your Editorial Manager account:*** [***https://www.youtube.com/watch?v=_xcclfuvtxQ***](https://www.youtube.com/watch?v=_xcclfuvtxQ)***.***

Thank you for your suggestion. The corresponding author has registered an ORCID iD (account no.: 0000-0001-5201-9529) and linked it to PLOS ONE author information system.

***4. We note that Figure 4 in your submission contain map images which may be copyrighted. All PLOS content is published under the Creative Commons Attribution License (CC BY 4.0), which means that the manuscript, images, and Supporting Information files will be freely available online, and any third party is permitted to access, download, copy, distribute, and use these materials in any way, even commercially, with proper attribution. For these reasons, we cannot publish previously copyrighted maps or satellite images created using proprietary data, such as Google software (Google Maps, Street View, and Earth). For more information, see our copyright guidelines: http://journals.plos.org/plosone/s/licenses-and-copyright.***

***We require you to either (1) present written permission from the copyright holder to publish these figures specifically under the CC BY 4.0 license, or (2) remove the figures from your submission:***

We appreciate for your nice advice. In the revised manuscript, we have removed Figure 4.

***5. In your Data Availability statement, you have not specified where the minimal data set underlying the results described in your manuscript can be found. PLOS defines a study's minimal data set as the underlying data used to reach the conclusions drawn in the manuscript and any additional data required to replicate the reported study findings in their entirety. All PLOS journals require that the minimal data set be made fully available. For more information about our data policy, please see*** [***http://journals.plos.org/plosone/s/data-availability***](http://journals.plos.org/plosone/s/data-availability)***.***

***Upon re-submitting your revised manuscript, please upload your study’s minimal underlying data set as either Supporting Information files or to a stable, public repository and include the relevant URLs, DOIs, or accession numbers within your revised cover letter. For a list of acceptable repositories, please see*** [***http://journals.plos.org/plosone/s/data-availability#loc-recommended-repositories***](http://journals.plos.org/plosone/s/data-availability#loc-recommended-repositories)***. Any potentially identifying patient information must be fully anonymized.***

***Important: If there are ethical or legal restrictions to sharing your data publicly, please explain these restrictions in detail. Please see our guidelines for more information on what we consider unacceptable restrictions to publicly sharing data:*** [***http://journals.plos.org/plosone/s/data-availability#loc-unacceptable-data-access-restrictions***](http://journals.plos.org/plosone/s/data-availability#loc-unacceptable-data-access-restrictions)***. Note that it is not acceptable for the authors to be the sole named individuals responsible for ensuring data access.***

***We will update your Data Availability statement to reflect the information you provide in your cover letter.***

Thank you for your advice. **All relevant data are within the manuscript**. We have added the Data Availability statement in the revised cover letter.

***6. Please review your reference list to ensure that it is complete and correct. If you have cited papers that have been retracted, please include the rationale for doing so in the manuscript text, or remove these references and replace them with relevant current references. Any changes to the reference list should be mentioned in the rebuttal letter that accompanies your revised manuscript. If you need to cite a retracted article, indicate the article’s retracted status in the References list and also include a citation and full reference for the retraction notice.***

Thank you for your suggestion. We have carefully reviewed the reference list to ensure that it is complete and correct. Please note that we removed Reference (37) from the original manuscript since we removed Figure 4.

***Addressing the comments of Reviewer #1***

***Author present an interesting integer linear programming model for the rail freight service network design problem with the example for China railway system.
In order to be eligible for the publication in Journal the author should more clearly and precisely elaborate on the innovative approach presented in this paper, i.e. how is this model improvement related to the existing and previous models used for this type of problems.***

Thank you for your advice. The rail freight service network design problem is a hot research topic during the last decades. The network flow model is widely adopted to mathematically describe the problem, such as Crainic and Rousseau (1986)^[1]^, Ahuja et al. (1993)^[2]^ and Lulli et al. (2011)^[3]^. However, these researches are mainly conducted in the context of the North American and European freight railway transportation systems. Although the decision problems are similar in nature, there are several differences among Chinese, North American and European freight railway transportation companies, both in the way of managing operations and in the way of handling tactical planning. Consequently, mathematical models developed for the European and North American systems may not be appropriate for the Chinese scenario. To bridge the research gap, this paper proposes a model to capture the unique feature in China railway system, i.e. the tree-shaped path, which is not carefully examined in other railway systems. Though some Chinese researchers try to model the tree-shaped path constraint, most of the models are non-linear (see, for example, Lin et al. (2020)^[4]^) and are difficult to solve to optimum even for small or medium scale instance. Our paper proposes an integer linear programming (ILP) that is able to solve the real-life problem with a scale of 139 stations, 473 alternative train services and 562 shipments to global optimum within a reasonable time frame using a state-of-the-art optimization solver.

***Addressing the comments of Reviewer #2***

***In this paper is proposed an integer linear programming model for the rail freight service network design problem with aim to minimize the overall costs in the context of the China railway system.***

***Тhe author emphasized that problem of rail freight service network design in North American and European freight railway transportation systems are similar in nature with Chines, but the paper only provides a parallel with the American freight transportation system (lines 82-87). Author also mentions on several occasions that problem of rail freight service network design are similar in Europe, but there are no additional explanations of main feature of these problem in Europe (about technology, operation, train routing, wagon management, etc). It would be very useful to add observations and papers related to the problems of design rail freight service and rail network design in the European context. This will support/strengthen the first contribution of this paper (lines 97- 101).***

We appreciate for your suggestion. To the best of our knowledge, there are two main differences between Chinese and European railway systems. As stated in the book *Theory and Methodology for Railway Train Operation Organization of the Planning Type* (in Chinese)^[5]^, the biggest difference between Chinese and European railway systems is the train operation organization strategy. The Chinese railway system belongs to the organizing type while the European railway system belongs to the planning type. The planning type is service quality oriented, which means the dispatch of train service is strictly depends on the train timetable. However, in the organizing type (quantity oriented), a train service cannot be dispatched if the number of loaded wagons does not reach a predefined threshold (e.g., 50 wagons). This means that sometimes a train no. will not be assigned with a real train service. Moreover, as the high-speed railway develops rapidly in China during the last decades, a large number of passenger train services transfer from normal-speed rail lines to high-speed rail lines. Consequently, the network capacity becomes much more sufficient than before, whereas most passenger trains and freight trains share the same rail lines in European railway systems and the network capacity is hence a hard constraint in the system^[6]^. The above two differences between Chinese and European railway systems result in the differences in mathematical formulation on the aspects of the train length constraint and network capacity constraint, etc.

***There is lack of some element’s notations. For example, what I, J, C denotes. Please, the author should double-check all the notations (lines 187-193).***

We appreciate for your suggestion. We have double-checked all the notations and added explanations for each symbol used in this paper. Please see the revised manuscript for detail.

***When explaining the modeling of the problem, the author introduces limitations in transportation time. However, it cannot be determined whether these are time restrictions due to the applied technology or whether they are restrictions due to network capacity (lines 213-217).***

Thank you for your comment. In this paper, the transportation time limitation constraint is set for the express goods, which are generally transported by express train services. In China railway practice, on one hand, express train services are usually dispatched prior to non-express train services; on the other hand, the express train services accounts for a very low share in the overall railway freight train services. These mean that the network capacity is sufficient for the express train services. Therefore, the transportation time is restricted by applied technology (i.e., Constraint (4)) rather than the network capacity.

We appreciate for Editors/Reviewers’ warm work earnestly, and hope that the correction will meet with approval.

Once again, thank you very much for your comments and suggestions.

Best regards,

Xiaoyu Xin

**Affiliation:** School of Traffic and Transportation, Beijing Jiaotong University

**E-mail:** [19114058@bjtu.edu.cn](mailto:19114058@bjtu.edu.cn)

1. Crainic, T. G., & Rousseau, J. M. (1986). Multicommodity, multimode freight transportation: A general modeling and algorithmic framework for the service network design problem. Transportation Research Part B: Methodological, 20(3), 225-242.
2. Ahuja, R. K., Magnanti, T. L., Orlin, J. B. (1993). Network flows: Theory, algorithms, and applications. Prentice Hall, Englewood Cliffs, NJ.
3. Lulli, G., Pietropaoli, U., & Ricciardi, N. (2011). Service network design for freight railway transportation: the Italian case. Journal of the Operational Research Society, 62(12), 2107-2119.
4. Lin, B., Zhao, Y., & Lin, R. (2020). Optimization for courier delivery service network design based on frequency delay. Computers & Industrial Engineering, 139, 106-144.
5. Siji Hu. (2017). Theory and Methodology for Railway Train Operation Organization of the Planning Type[M]. Beijing: China Railway Publishing House.
6. Clausen U, Voll R. A comparison of North American and European railway systems[J]. European Transport Research Review, 2013, 5(3): 129-133.
