# Supplementary material for: Service network design for freight railway transportation: The Chinese case
Source: PLoS One. 2022 Dec 1;17(12):e0276671. doi: 10.1371/journal.pone.0276671 (PMC9714731; doi:10.1371/journal.pone.0276671)
Supplement: Attachment — Submitted filename: Response to Reviewers.docx [file pone.0276671.s002.docx]

**Dear Prof. Dragan Pamucar for our paper PONE-D-22-11121R1:**

The authors sincerely thank you for the comments and suggestions which contribute to enhancing the quality of the paper. We have addressed the editor’s and reviewers’ comments and suggestions as well as the journal requirements in the revised paper. Each comment or suggestion is shown below in *Italic* followed with our action to modify the paper accordingly.

***Addressing the Journal Requirements***

***Please review your reference list to ensure that it is complete and correct. If you have cited papers that have been retracted, please include the rationale for doing so in the manuscript text, or remove these references and replace them with relevant current references. Any changes to the reference list should be mentioned in the rebuttal letter that accompanies your revised manuscript. If you need to cite a retracted article, indicate the article’s retracted status in the References list and also include a citation and full reference for the retraction notice.***

Thank you for your suggestion. We have carefully reviewed the reference list to ensure that it is complete and correct. Please note that we added Reference (37) and (38) into the revised paper, accompanying our revisions that suggested by the reviewer.

***Addressing the comments of Reviewer #2***

***Thank you very much for the explanation you gave, but you did not improve the paper considering those responses to the comments.***

***I suggest that you include a detailed explanation about transport time in the paper, because it precisely indicates the difference between the European and Chinese freight systems. Also expand the explanation of the difference between the European and Chinese systems in the text.***

We appreciate for your suggestion. In the revised paper, we have included a detailed explanation about transport time. Explanation of the difference between the European and Chinese systems has also been added into the text of the revised paper.

***Additionaly, for comparisons between the optimal solution and a manual solution are used results of overall costs and solution time (lines 356-356). For solution time, operational times for defining train routes by highly experienced dispatchers (skill, competence) and computer speed (depends on the type of computer, software solution and amount of data) are compared. It is not clear what is included in overall cost figure. Are these only transportation costs or they are also other costs? Tariffs are the same because the same type and quantity of goods are transported. The author should explain in the text and strengthen results in this sense.***

Thank you for your advice. The overall costs consist of train costs and car costs. The train cost includes the costs of depreciation, maintenance, power, tool and engine drivers, and it is associated with train service type (class), train service itinerary length, operation costs of train coupling/uncoupling (i.e., handling costs), etc. The car cost includes both car depreciation and maintenance, and it is associated with itinerary length, goods volume, goods category, etc. We have explained the detailed meaning of the overall cost figure in our revised manuscript.

We appreciate for Editors/Reviewers’ warm work earnestly, and hope that the correction will meet with approval.

Once again, thank you very much for your comments and suggestions.

Best regards,

Xiaoyu Xin

**Affiliation:** School of Traffic and Transportation, Beijing Jiaotong University

**E-mail:** [19114058@bjtu.edu.cn](mailto:19114058@bjtu.edu.cn)
